# Clinical characteristics and survival of pulmonary arterial hypertension with or without interstitial lung disease in systemic sclerosis

**DOI:** 10.1186/s13075-023-03059-x

**Published:** 2023-05-12

**Authors:** Jessica L. Fairley, Dylan Hansen, Laura Ross, Susanna Proudman, Joanne Sahhar, Gene-Siew Ngian, Jennifer Walker, Lauren V. Host, Kathleen Morrisroe, Diane Apostolopoulous, Nava Ferdowsi, Michelle Wilson, Maryam Tabesh, Wendy Stevens, Mandana Nikpour, Australian Scleroderma Interest Group, Helen Cooley, Lucy Croyle, Catherine Hill, Lauren Host, Sue Lester, Gabor Major, Peter Nash, Maureen Rischmueller, Janet Roddy, Gemma Strickland, Tien Tay, Kathleen Tymms, Peter Youssef

**Affiliations:** 1 The University of Melbourne at St. Vincent’s Hospital, 41 Victoria Parade Fitzroy, Melbourne, VIC 3065 Australia; 2 St. Vincent’s Hospital Melbourne, Melbourne, VIC Australia; 3 University of Adelaide, Adelaide, South Australia Australia; 4 Royal Adelaide Hospital, Adelaide, South Australia Australia; 5 Monash Health, Melbourne, VIC Australia; 6 Monash University, Melbourne, VIC Australia; 7 Fiona Stanley Hospital, Perth, WA Australia

**Keywords:** Pulmonary hypertension, Interstitial lung disease, Survival, Quality of life

## Abstract

**Objectives:**

To describe the clinical phenotype and prognosis of people in the Australian Scleroderma (SSc) Cohort Study with pulmonary arterial hypertension (PAH) with or without interstitial lung disease (ILD).

**Methods:**

Participants meeting ACR/EULAR criteria for SSc were divided into four mutually exclusive groups: those meeting criteria for PAH (PAH-only), ILD (ILD-only), concurrent PAH and ILD (PAH-ILD) or neither PAH nor ILD (SSc-only). Logistic or linear regression analyses were used for associations between clinical features, health-related quality of life (HRQoL) and physical function. Survival analysis was performed using Kaplan–Meier estimates and Cox-regression modelling.

**Results:**

Of 1561 participants, 7% fulfilled criteria for PAH-only, 24% ILD-only, 7% PAH-ILD and 62% SSc-only. People with PAH-ILD were more frequently male, with diffuse skin involvement, higher inflammatory markers, older age of SSc onset and higher frequency of extensive ILD than the cohort overall (*p* < 0.001). People of Asian race more frequently developed PAH-ILD (*p* < 0.001). People with PAH-ILD or PAH-only had worse WHO functional class and 6-min-walk-distance than ILD-only (*p* < 0.001). HRQoL scores were worst in those with PAH-ILD (*p* < 0.001).

Survival was reduced in the PAH-only and PAH-ILD groups (*p* < 0.01). Multivariable hazard modelling demonstrated the worst prognosis in extensive ILD and PAH (HR = 5.65 95% CI 3.50–9.12 *p* < 0.01), followed by PAH-only (HR = 4.21 95% CI 2.89–6.13 *p* < 0.01) and PAH with limited ILD (HR = 2.46 95% CI 1.52–3.99 *p* < 0.01).

**Conclusions:**

The prevalence of concurrent PAH-ILD in the ASCS is 7%, with poorer survival in those patients with PAH-ILD compared to ILD or SSc alone. The presence of PAH confers a poorer overall prognosis than even extensive ILD; however, further data are required to better understand the clinical outcomes of this high-risk patient group.

**Supplementary Information:**

The online version contains supplementary material available at 10.1186/s13075-023-03059-x.

## Key messages

- Pulmonary arterial hypertension and interstitial lung disease occur concurrently in 7% of this SSc cohort.
- Survival in cohorts with PAH-ILD was poorer with increasing severity of ILD.
- Physical function and health-related quality of life were worst in those with PAH and ILD.

## Background

Pulmonary arterial hypertension (PAH) and interstitial lung disease (ILD) are leading causes of death in people with systemic sclerosis (SSc) [1]. In SSc, pulmonary hypertension (PH) can have multiple causes; 60–70% of PH is thought to be group I PAH, whereas 20% may be group III PH due to hypoxaemic lung disease, and 10–20% group II PH due to cardiac disease [2, 3]. Up to 75–90% of individuals with SSc may demonstrate some degree of ILD on high-resolution computed tomography (HRCT) [4, 5], whilst lifetime prevalence of SSc-PAH is approximately 12% [6, 7]. PAH and ILD have been generally viewed as independent manifestations of SSc; however, given the high prevalence of both complications, there is increasing interest in describing individuals who develop both pre-capillary PAH and ILD concurrently [8–10]. Existing studies are characterised by modest population size [8, 11, 12] or are cross-sectional in nature thereby lacking longitudinal data [9].

The prognosis of people who develop concurrent PAH and ILD appears to be particularly poor [11]. PH-specific therapy has progressed rapidly and is associated with improved survival in SSc-PAH [6]. However, in precapillary PAH and ILD, the situation is less clear, particularly given concerns about potential for accelerated disease progression with use of endothelin receptor antagonists (ERA) in idiopathic pulmonary fibrosis [13]. Exacerbation of ventilation-perfusion mismatch may also exacerbate hypoxia in PAH combined with other parenchymal lung diseases [14]. A better understanding of people with SSc with PAH and ILD is critical in optimising management and identifying opportunities for improved survival.

Accordingly, this study describes the clinical phenotype, prognosis and quality of life of people with concurrent PAH and ILD in the Australian Scleroderma Cohort Study (ASCS). We aimed to define a clinical phenotype of concurrent PAH and ILD and compare survival and quality of life between individuals with PAH and ILD, compared to those with PAH or ILD alone.

## Participants and methods

### Participants

Participants were recruited from the ASCS, a multicentre Australian study of SSc. The ASCS has been approved by all Human Research Ethics Committees of participating sites. Written informed consent was obtained from all participants prior to the collection of any study data. This study is conducted in accordance with the Declaration of Helsinki.

### Selection of groups

We included participants who met American College of Rheumatology/European League against Rheumatism (ACR/EULAR) criteria for SSc between 2007 and October 2019 [15]. All ASCS participants were screened annually for PH with both transthoracic echocardiography (TTE) and pulmonary function tests (PFT). Definitive diagnostic testing with right heart catheterisation (RHC) and/or HRCT of the chest was performed if either TTE or PFT were abnormal or in response to suggestive clinical signs or symptoms. Results considered highly suspicious for precapillary PAH warranting RHC referral were a right ventricular systolic pressure (RVSP) of ≥ 50 mmHg. Symptomatic participants recording an RVSP of 40–50 mmHg or a diffusing capacity for carbon monoxide (DLCO) of < 50% predicted with no alternate explanation were also considered to be at high risk for precapillary PAH and referred for RHC. Participants with an RSVP of 30–40 mmHg with a DLCO > 50% predicted were considered to be at moderate risk of PAH and assessed by the clinician on a case-by-case basis. PFT parameters were considered abnormal at < 80 percent-predicted or according to local laboratory protocols.

PAH was defined by RHC findings consistent with either the revised PAH classification criteria [16] (mean pulmonary artery pressure (mPAP) > 20 mmHg, pulmonary vascular resistance (PVR) > 3 Wood units and a pulmonary arterial wedge pressure (PAWP) ≤ 15 mmHg) or previous classification criteria [17] (mPAP ≥ 25 mmHg, PAWP < 15 mmHg), as not all ASCS participants had PVRs recorded on early RHC studies. A small number of participants had a medical contraindication to RHC (e.g. repeated failed procedures due to anatomic difficulties) or pretreatment RHC was unavailable but had highly suggestive echocardiography and were treated as having PAH and thus were included by study physicians. Chest HRCT was not routinely performed but instead performed at physician discretion in response to clinical symptoms/signs or abnormal PFT. ILD was considered present if there were typical interstitial findings on HRCT of the chest. Severity of ILD was defined by the extent of radiological involvement as either limited (< 20% HRCT involvement) or extensive (> 30% HRCT involvement) [18]. Where HRCT extent was 20–30%, percent-predicted forced vital capacity (FVC) of < 70% was used to classify patients as extensive ILD or FVC ≥ 70% as limited ILD.

We divided participants into four mutually exclusive groups: PAH alone (PAH-only), ILD alone (ILD-only), both precapillary PAH and ILD (PAH-ILD) or neither PAH nor ILD (SSc-only). Participants were included in the PAH group if they met the above criteria for PAH without history of ILD, in the ILD-only group if they met criteria for ILD without history of PAH and in the PAH and ILD group if they met criteria for both conditions. Participants were included in the SSc-only group if they did not meet criteria for either condition. Participants reviewed by study physicians with borderline pulmonary pressures not deemed to have precapillary PH (e.g. those with group II PH due to left heart disease) were excluded from the study.

### Autoantibody testing

Autoantibody status was defined by a positive result according to the local laboratory reference range. Anti-nuclear antibody (ANA) was detected using indirect immunofluorescence, with positivity defined as titres ≥ 1:80. Extractable nuclear antigens (ENA) and RNA-polymerase 3 antibodies were detected using ELISA, immunoblot or a combination of these (based on local laboratory commercial testing kits).

### Data collection

Demographic and disease data were prospectively collected annually in a standardised format. Disease manifestations and autoantibody results were considered present if they had ever been reported from SSc diagnosis. Disease onset and duration were defined by the date of onset of the first non-Raynaud’s phenomenon disease manifestation. The LeRoy criteria were used to determine disease subtype (diffuse (dcSSc) or limited (lcSSc)) [19]. Overlap conditions were defined by the treating physician if there were clinical features of another connective tissue disease present, although it was not mandated that participants independently fulfil diagnostic criteria for these conditions. Scleroderma renal crisis (SRC) was diagnosed by the presence of at least two of three criteria: new-onset hypertension with no alternate cause, unexplained rise in serum creatinine or microangiopathic haemolytic anaemia. Endoscopy was used to diagnose gastric antral vascular ectasia (GAVE) and reflux oesophagitis. Patient-reported outcomes (PRO) about physical function and health-related quality of life (HRQoL) were collected annually. To measure comorbidity burden, we calculated a modified Charlson Comorbidity Index (CCI) score [15]. A list of included items is provided in Supplementary Table S1; data for some variables (including hemiplegia, HIV/AIDS and dementia) were excluded as these data are not collected as part of the ASCS protocol. A CCI score ≥ 4 was defined as a significant comorbidity burden [16], with the highest available score being 19.

### Statistical analysis

Characteristics of study participants are presented as mean (standard deviation (SD)) for normally distributed continuous variables, median (interquartile range (IQR)) for non-normally distributed continuous variables and as a number (percentage) for discrete variables. Comparisons between groups were performed using analysis of variance and covariance (ANOVA) for normally distributed continuous variables, the Kruskal–Wallis rank test for non-normally distributed continuous variables and the chi-squared test for discrete variables.

Survival analysis was performed using the endpoint of all-cause mortality. Kaplan–Meier survival curves and the Wilcoxon test were used to estimate survival from SSc onset. A multivariable Cox proportional hazards regression analysis was used to determine multivariate predictors of mortality. Covariates were chosen for the multivariate analysis if they were either clinically relevant or statistically significant on univariate analysis (*p* < 0.05) and did not violate the proportional hazards assumption. The results are reported as hazard ratios (HR) with accompanying 95% confidence intervals (CI). Generalised estimating equations (GEE) using an exchangeable correlation structure were used to model longitudinal data involving repeated measures. PRO data were compared to ASCS or population average values as specified. All statistical analyses were performed using STATA 15.1 (Statacorp LP, College Station, TX, USA).

## Results

### Demographics of the cohort

**Fig. 1:**
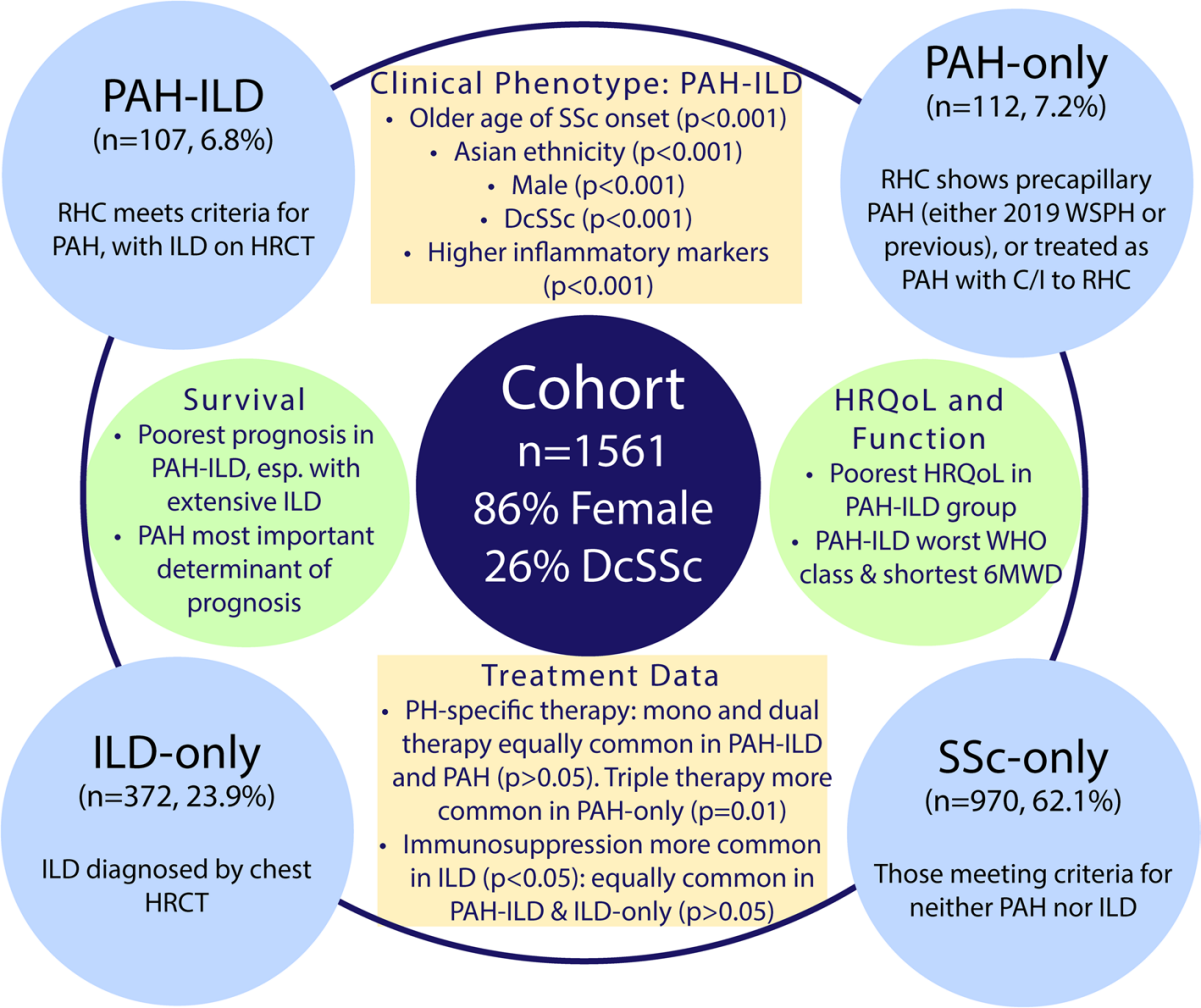
Key findings. 6MWD, six-minute walk distance; C/I, contraindication; dcSSc, diffuse cutaneous systemic sclerosis; ILD, interstitial lung disease; PAH, pulmonary arterial hypertension); PH, pulmonary hypertension; RHC, right heart catheter; SSc, systemic sclerosis; WHO, World Health Organization; WSPH, World Society of Pulmonary Hypertension

One-thousand five-hundred and sixty-one individuals fulfilled the inclusion criteria (Supplementary Figure S1, Table 1), of whom 1349 participants (86%) were female, 1157 (74%) had lcSSc and 404 (26%) dcSSc. One-hundred and seven (6.9%) met criteria for both PAH and ILD (PAH-ILD group), 112 (7.2%) PAH alone (PAH-only group), 372 (23.9%) ILD alone (ILD-only group), and 970 (62.1%) neither condition (SSc-only group) (Fig. 1).

**Table 1:** Baseline characteristics of the study population

**Variable**		**Disease group**^**a**^ ***n* (%) or mean**				
	**Cohort overall** **(*n* = 1561)**	**PAH-ILD** **(*n* = 107)**	**PAH-only** **(*n* = 112)**	**ILD-only** **(*n* = 372)**	**SSc-only** **(*n* = 970)**	***p*-value**
**Female**	1349 (86.4%)	83 (77.6%)	98 (87.5%)	308 (82.8%)	860 (88.7%)	0.001
**Race**						
Caucasian	1345 (90.9%)	90 (88.2%)	98 (94.2%)	296 (83.2%)	861 (93.9%)	< 0.001
Asian	80 (5.4%)	9 (8.8%)	2 (1.9%)	42 (11.8%)	27 (2.9%)	
Aboriginal-Torres Strait Islander	17 (1.2%)	1 (1.0%)	2 (1.9%)	6 (1.7%)	8 (0.9%)	
Hispanic	12 (0.8%)	0 (0.0%)	2 (1.9%)	2 (0.6%)	8 (0.9%)	
Other	25 (1.7%)	2 (2.0%)	0 (0.0%)	10 (2.8%)	13 (1.4%)	
**Age of SSc onset (years)**^**b**^	47.1 (36.2–56.4)	50.5 (42.6–60.2)	51.2 (41.4–61.5)	47.1 (35.0–56.2)	45.9 (35.6–55.2)	< 0.001
**Disease duration at recruitment (years)**^**b**^	7.5 (2.5–15.9)	7.8 (2.8–17.9)	10.8 (4.2–20.5)	6.4 (2.1– 14.3)	7.8 (2.5–15.7)	0.017
**Diffuse skin disease**	404 (25.9%)	36 (34.0%)	13 (11.6%)	138 (37.0%)	217 (22.4%)	< 0.001
**Overlap CTD**	127 (13.4%)	6 (8.0%)	5 (7.7%)	37 (15.0%)	79 (14.1%)	0.212
Rheumatoid arthritis	54 (3.5%)	1 (0.9%)	3 (2.7%)	15 (4.0%)	35 (3.6%)	0.36
SLE	18 (1.2%)	0 (0.0%)	1 (0.9%)	6 (1.6%)	11 (1.1%)	0.61
Polymyositis	26 (1.7%)	2 (1.9%)	0 (0.0%)	11 (3.0%)	13 (1.3%)	0.22
Sjögren syndrome	37 (2.4%)	4 (3.7%)	0 (0.0%)	10 (2.7%)	23 (2.4%)	0.29
**Follow-up (years)**	3.5 (1.0–7.1)	4.0 (2.0–7.7)	3.6 (1.0–6.9)	4.1 (1.2–8.2)	3.3 (1.0–6.7)	0.003
**Deceased**	232 (14.9%)	54 (50.5%)	53 (47.3%)	60 (16.2%)	65 (6.7%)	< 0.001
Ever smoked*	752 (48.6%)	46 (43.8%)	54 (50.0%)	154 (41.6%)	498 (51.6%)	0.008
Comorbidities (CCI score)*	2 (1–3)	3 (2–4)	3 (2–4)	2 (1–3)	2 (1–3)	< 0.001
**Antibody profile**						
**ANA centromere positive** (*n* = 1486)	694 (46.7%)	26 (25.7%)	86 (79.6%)	61 (17.2%)	521 (56.5%)	< 0.001
**ENA subtype**						
Anti-Scl-70 (*n* = 1463)	230 (15.7%)	17 (16.8%)	1 (1.0%)	135 (38.5%)	77 (8.5%)	< 0.001
Anti-RNP (*n* = 1461)	96 (6.6%)	6 (5.9%)	7 (6.7%)	31 (8.9%)	52 (5.8%)	0.256
Anti-Ro60 (*n* = 1459)	127 (8.7%)	17 (16.7%)	11 (10.7%)	44 (12.6%)	55 (6.1%)	< 0.001
Anti-La (*n* = 1456)	22 (1.5%)	3 (3.0%)	2 (1.9%)	6 (1.7%)	11 (1.2%)	0.533
**Anti-dsDNA** (*n* = 1235)	92 (7.3%)	6 (6.3%)	6 (6.7%)	25 (7.8%)	55 (7.3%)	0.964
**RNA polymerase III** (*n* = 1103)	151 (13.7%)	17 (21.0%)	4 (5.0%)	33 (12.0%)	97 (14.5%)	0.021
**ANCA** (*n* = 1396)	221 (15.8%)	26 (27.7%)	10 (10.3%)	78 (23.2%)	107 (12.3%)	< 0.001
**MPO-ANCA** (*n* = 1390)	25 (1.8%)	1 (1.1%)	0 (0.0%)	12 (3.6%)	12 (1.4%)	0.031
**PR3-ANCA** (*n* = 1390)	32 (2.3%)	4 (4.3%)	1 (1.0%)	9 (2.7%)	18 (2.1%)	0.433
**Antiphospholipid antibodies** (*n* = 1378)	360 (26.1%)	33 (34.4%)	32 (32.7%)	96 (29.3%)	199 (23.3%)	0.012

*Abbreviations*: *ANA* antinuclear antibody, *ANCA* anti-neutrophil cytoplasmic antibodies, *ASCS* Australian Scleroderma Cohort Study, *CCI* Charlson Comorbidity Index, *CCP* cyclic citrullinated peptide, *CTD* connective tissue disease, *dsDNA* double-stranded deoxyribonucleic acid, *ENA* extractable nuclear antigen, *ILD* interstitial lung disease, *MPO* myeloperoxidase, *n* number, *PAH* pulmonary arterial hypertension, *PR3* proteinase-3, *RNA* ribonucleic acid, *RNP* ribonucleoprotein, *SLE* systemic lupus erythematosus, *Sm* Smith, *SSc* systemic sclerosis

^a^Four mutually exclusive disease groups based on presence of concurrent PAH and ILD (PAH-ILD), PAH alone (PAH-only), ILD alone (ILD-only) or neither comorbidity (SSc-only)

^b^Time from first symptom other than Raynaud’s phenomenon

*Comorbidities defined by the modified Charlson Comorbidity Index Score

Those with PAH-ILD were more likely to be male (*p* = 0.001) with dcSSc (*p* < 0.001), whilst those with PAH-only were more likely to have lcSSc (*p* < 0.001) (Table 1). People of Asian ethnicity were more likely to have ILD-only or PAH-ILD (*p* < 0.001). Those with PAH-only or PAH-ILD were older at SSc onset (*p* < 0.001), with a longer SSc duration at recruitment in those with PAH-only (*p* = 0.017). ASCS follow-up was 3.3–4.1 years, slightly longer in those with PAH-ILD or ILD-only (*p* = 0.003). People with SSc-only or PAH-only were more likely to have smoked (*p* = 0.008). People in the PAH-only or PAH-ILD groups were more likely to have died (PAH-ILD 50.5%, PAH-only 47.3%, ILD-only 16.2%, SSc-only 6.7%, *p* < 0.001). Comorbidity burden as measured by the CCI score.

### Autoantibody profiles of the four disease groups

Anti-centromere positivity was most common in those with PAH-only, followed by SSc-only (*p* < 0.001; Table 1). Scl-70 positivity was most frequent in those with ILD-only and uncommon in PAH-only (*p* < 0.001). Anti-Ro60 positivity was most common in those with PAH-ILD (*p* < 0.001), as were RNA polymerase-3 antibodies (*p* = 0.018) and antiphospholipid antibodies (*p* = 0.012). ANCA positivity was most common in those with ILD (ILD-only or PAH-ILD; *p* < 0.001).

### Clinical characteristics and organ involvement

#### PH- and ILD-specific data

Those with PAH-ILD most frequently had extensive ILD (PAH-ILD 44.9%, ILD-only 23.1%, *p* < 0.001), whilst limited ILD was more frequent in those with ILD-only (PAH-ILD 43.6%, ILD-only 60.9%, *p* < 0.001) (Table 2). Lowest six-minute walk distance (6MWD) occurred in those with PAH-ILD or PAH-only (*p* < 0.001). WHO functional class III or IV dyspnoea occurred in more than 90% of those with PAH-ILD (PAH-ILD 91.4%, PAH-only 85.8%, ILD-only 36.8%, SSc-only 15.0%, *p* < 0.001). Percent-predicted FVC was lowest in those with PAH-ILD (*p* < 0.001), as was percent-predicted DLCO (*p* < 0.001). People with PAH-only or PAH-ILD were more likely to have right ventricular (RV) dysfunction (*p* < 0.001). On RHC at PAH diagnosis, right atrial pressure, mPAP, PVR and PAWP were similar between those with PAH-only and PAH-ILD (*p* > 0.05), as were cardiac output and cardiac index (*p* > 0.05).

**Table 2:** SSc disease features and treatment of the study population

Variable	Disease group^a^ *n* (%) or mean				
	**PAH-ILD** **(*n* = 107)**	**PAH-only** **(*n* = 112)**	**ILD-only** **(*n* = 372)**	**SSc-only** **(*n* = 970)**	***p*-value**
**PAH- and ILD-specific features**					
**HRCT performed**	107 (100%)	81 (72.3%)	372 (100%)	307 (31.7%)	< 0.001
**ILD severity**^**b**^					< 0.001^#^
Limited	55 (51.4%)	N/A	244 (65.6%)	N/A	
Extensive	48 (44.9%)	N/A	86 (23.1%)	N/A	
Missing	4 (3.7%)	N/A	42 (11.3%)	N/A	
**Pulmonary function testing**^c^					
FVC (lowest)	63.0 (51.0–83.0)	85.5 (73.0–97.5)	74.4 (62.0–90.0)	95.0 (84.0–106.0)	< 0.001
DLCO (lowest, %)	33.4 (27.0–43.0)	43.2 (33.0–51.9)	51.9 (40.5–63.0)	70.1 (59.2–81.8)	< 0.001
Six-minute walk test performed	101 (94.4%)	104 (92.9%)	274 (73.7%)	657 (67.7%)	< 0.001
Six-minute walk distance (lowest, m)	280.0 (180.0–384.0)	295.5 (209.0–364.0)	444.0 (360.0–515.0)	465.0 (395.0–536.0)	< 0.001
**WHO functional class (dyspnoea; highest recorded)**^c^					
Class I	1 (1.0%)	3 (2.8%)	91 (25.4%)	436 (46.9%)	< 0.001
Class II	8 (7.8%)	12 (11.3%)	136 (37.9%)	354 (38.1%)	
Class III	58 (55.8%)	67 (63.2%)	116 (32.3%)	131 (14.1%)	
Class IV	37 (35.6%)	24 (22.6%)	16 (4.5%)	8 (0.9%)	
**Echocardiography**					
RVSP (median, mmHg)	47 (39–61.5)	53 (39.5–77.5)	31.5 (27–37)	29 (26–33.5)	< 0.001, 0.052^
**RV dysfunction**^c^					
Normal RV function	44 (42.3%)	50 (45.9%)	328 (89.9%)	895 (95.6%)	< 0.001
Mild	30 (28.9%)	23 (21.1%)	27 (7.4%)	37 (4.0%)	
Moderate	14 (13.5%)	16 (14.7%)	7 (1.9%)	3 (0.3%)	
Severe	16 (15.4%)	20 (18.3%)	3 (0.8%)	1 (0.1%)	
**Right heart catheter (RHC) at PAH diagnosis or first-recorded value in ILD-only and SSc-only groups**					
RHC performed^d^	101 (95.3%)	111 (99.1%)	47 (12.6%)	51 (5.3%)	< 0.001
RAP (mmHg)	8 (5–10)	8 (6–10)	5 (3–7)	6 (4–10)	0.493^; 0.174^&^
PAWP (mmHg)	10.5 (8–13)	10.5 (8–13)	9 (7–14)	9.5 (7–18)	0.690^; 0.525^&^
Mean PAP (mmHg)	32 (28–41)	33 (27–43.5)	19 (16–23)	19 (15–23)	0.630^; 0.898^&^
PVR (Wood units)	4.5 (2.9–6.8)	4.1 (2.9–6.6)	1.9 (1.3–2.3)	2 (1.2–2.6)	0.919^; 0.899^&^
Cardiac output (L/min)	5.2 (4.1–6.2)	5.1 (4.1–6.3)	5.2 (4.8–5.5)	5.5 (4.7–6.0)	0.976^; 0.150^&^
Cardiac index (L/min/m^2^)	2.8 (2.5–3.4)	2.5 (2.1–3.4)	2.9 (2.7–3.2)	3.0 (2.7–3.5)	0.176^; 0.430^&^
**Other SSc disease features and treatment**					
**Disease features**					
Raynaud phenomenon^c^	107 (100%)	111 (99.1%)	367 (98.7%)	960 (99.0%)	0.682
Modified Rodnan skin score (highest)^c^	10.0 (5.0–19.0)	9.0 (5.0–15.0)	10.0 (6.0–19.0)	7.0 (4.0–14.0)	< 0.001
Digital ulcers^c^	70 (65.4%)	65 (58.0%)	209 (56.2%)	462 (47.6%)	< 0.001
Non-hand skin ulcers^c^	10 (9.4%)	12 (10.7%)	55 (14.8%)	69 (7.1%)	< 0.001
Calcinosis requiring antibiotics or surgery^c^	7 (6.5%)	5 (4.5%)	13 (3.5%)	47 (4.9%)	< 0.001
Synovitis^c^	44 (41.1%)	35 (31.3%)	157 (42.2%)	364 (37.5%)	0.148
Myositis^c^	4 (3.7%)	1 (0.9%)	17 (4.6%)	17 (1.8%)	0.014
Dry eyes^c^	66 (61.7%)	82 (73.2%)	231 (62.1%)	608 (62.7%)	< 0.001
Dry mouth^c^	84 (78.5%)	92 (82.1%)	276 (74.2%)	679 (70.0%)	< 0.001
Reflux symptoms^c^	85 (79.4%)	83 (74.1%)	321 (86.3%)	786 (81.0%)	0.022
Dysphagia^c^	56 (52.3%)	58 (51.8%)	186 (50.0%)	417 (43.0%)	0.008
Vomiting^c^	30 (28.0%)	29 (25.9%)	82 (22.0%)	194 (20.0%)	0.016
GAVE^c^	9 (8.4%)	15 (13.4%)	33 (8.9%)	91 (9.4%)	0.512
Scleroderma renal crisis^c^	7 (6.5%)	1 (0.9%)	7 (1.9%)	28 (2.9%)	0.040
Highest CRP^c^	11.5 (5.3–25)	7.8 (5–20)	7 (4–15)	5 (2.7–9)	< 0.001
Highest ESR^c^	35.0 (22.0–51.0)	28.0 (15.0–49.0)	28.0 (16.0–42.5)	19.0 (10.0–31.0)	< 0.001
Serum CK (highest recorded)^c^	112 (61–178)	88 (61–131)	113 (75–175)	100 (72–146)	0.002
Haemoglobin (lowest recorded)^c^	116 (106–131)	116 (106–128)	124 (113–132)	127 (119–135)	< 0.001
**PAH-specific treatments**					
Any vasodilator therapy^c^	97 (90.7%)	110 (98.2%)	0 (0%)	0 (0%)	< 0.001; 0.014^
PDE5^c^	64 (59.8%)	67 (59.8%)	0 (0%)	0 (0%)	< 0.001; 0.999^
ERA^c^	87 (81.3%)	104 (92.9%)	0 (0%)	0 (0%)	< 0.001; 0.016^
Prostacyclin analogues^c^	6 (5.6%)	14 (12.5%)	0 (0%)	0 (0%)	< 0.001; 0.080^
Monotherapy^c^	50 (46.7%)	61 (54.5%)	0 (0%)	0 (0%)	< 0.001; 0.252^
ERA^c^	60 (56.1%)	77 (68.8%)	0 (0%)	0 (0%)	< 0.001; 0.053^
PDE5^c^	21 (19.6%)	21 (18.8%)	0 (0%)	0 (0%)	< 0.001; 0.869^
PCA^c^	2 (1.9%)	2 (1.8%)	0 (0%)	0 (0%)	< 0.001; 0.963^
Dual therapy^c^	46 (43.0%)	40 (35.7%)	0 (0%)	0 (0%)	< 0.001; 0.270^
ERA and PDE5^c^	44 (41.1%)	39 (34.8%)	0 (0%)	0 (0%)	< 0.001; 0.337^
ERA and PCA^c^	1 (0.9%)	1 (0.9%)	0 (0%)	0 (0%)	0.006; 0.974^
PDE5 and PCA^c^	1 (0.9%)	0 (0.0%)	0 (0%)	0 (0%)	0.004; 0.305^
Triple therapy^c^	1 (0.9%)	9 (8.0%)	0 (0%)	0 (0%)	< 0.001; 0.012^
Lung transplantation^c^	1 (0.9%)	1 (0.9%)	4 (1.1%)	0 (0.0%)	0.019
**Other treatments**					
PPI^c^	91 (85.1%)	98 (87.5%)	326 (87.6%)	763 (78.7%)	< 0.001
H2 antagonist^c^	25 (23.6%)	26 (23.2%)	94 (25.3%)	219 (22.6%)	0.735
Promotility agents^c^	11 (10.4%)	14 (12.5%)	70 (18.8%)	152 (15.7%)	0.131
Prednisolone^c^	69 (64.5%)	30 (26.8%)	222 (59.7%)	354 (36.5%)	< 0.001; 0.299^#^
Methotrexate^c^	19 (17.9%)	9 (8.0%)	99 (26.6%)	231 (23.8%)	< 0.001; 0.076^#^
Azathioprine^c^	19 (17.8%)	1 (0.9%)	80 (21.5%)	38 (3.9%)	< 0.001; 0.429^#^
Mycophenolate^c^	26 (24.3%)	5 (4.5%)	107 (28.8%)	56 (5.8%)	< 0.001; 0.399^#^
Leflunomide^c^	2 (1.9%)	0 (0.0%)	5 (1.3%)	12 (1.2%)	0.609
Cyclophosphamide^c^	18 (16.8%)	5 (4.5%)	80 (21.5%)	26 (2.7%)	< 0.001; 0.314^#^
Hydroxychloroquine^c^	18 (17.0%)	15 (13.4%)	92 (24.7%)	220 (22.7%)	0.043
TNF-alpha inhibitors^c^	1 (0.9%)	1 (0.9%)	5 (1.3%)	7 (0.7%)	0.762
Rituximab^c^	2 (1.9%)	0 (0.0%)	12 (3.2%)	6 (0.6%)	0.001; 0.473^#^
Tocilizumab^c^	0 (0.0%)	0 (0.0%)	7 (1.9%)	4 (0.4%)	0.018; 0.155^#^
Abatacept^c^	0 (0.0%)	0 (0.0%)	3 (0.8%)	1 (0.1%)	0.120

*Abbreviations*: *CK* creatine kinase, *CRP* C-reactive protein, *DLCO* diffusing capacity for carbon monoxide, *ERA* endothelin receptor antagonist, *ESR* erythrocyte sedimentation rate, *FVC* forced vital capacity, *GAVE* gastric antral vascular ectasia, *H2* histamine receptor H2, *ILD* interstitial lung disease, *m* metres, *n* number, *PAH* pulmonary arterial hypertension, *PAP* pulmonary artery pressure, *PAWP* pulmonary arterial wedge pressure, *PDE5* phosphodiesterase type 5 inhibitor, *PPI* proton pump inhibitor, *PVR* pulmonary vascular resistance, *RHC* right heart catheter, *RV* right ventricle, *SSc* systemic sclerosis, *WHO* World Health Organization

^a^Four mutually exclusive disease groups based on presence of concurrent PAH and ILD (PAH-ILD), PAH alone (PAH-only), ILD alone (ILD-only) or neither comorbidity (SSc-only)

^b^ILD severity defined as limited if < 20% involvement on HRCT or 20–30% HRCT involvement with FVC ≥ 70%, or extensive if > 30% HRCT involvement, or 20–30% HRCT involvement and FVC < 70%

^c^Ever present from SSc diagnosis

^d^Right heart catheter study performed as clinically indicated

^*p*-value for comparison between the PAH-ILD and PAH-only groups

^#^*p*-value for comparison between the PAH-ILD and ILD-only groups

^&^*p*-value for comparison between the SSc-only and ILD-only groups

#### PAH-specific treatments

For treatment of PAH, ERA were most commonly prescribed, followed by phosphodiesterase type 5 inhibitors (PDE5i) and prostacyclin analogues (PCA). Overall, 98.2% of people with PAH-only and 90.7% of PAH-ILD received PH-specific medications (*p* = 0.014). Of those with PAH-ILD, 89.6% of those with extensive ILD received PH-specific medications compared to 92.7% of those with limited ILD (*p* = 0.573). Only 22 participants (1.5%) had used PH-specific medication prior to ASCS recruitment.

ERA were more commonly prescribed in PAH-only than PAH-ILD (PAH-ILD 82.1%, PAH-only 92.9%, *p* = 0.016), as were PCA although this did not reach statistical significance (PAH-ILD 5.7%, PAH-only 12.5%, *p* = 0.080). PDE5i were prescribed equally between the groups (PAH-ILD and PAH-only both 59.8%, *p* = 0.999). Monotherapy with PAH-specific treatment was more common than dual therapy, with no difference between PAH-only and PAH-ILD (monotherapy PAH-ILD 46.7%, PAH-only 54.5%, *p* = 0.252; dual PAH-ILD 43.0%, PAH-only 35.7%, *p* = 0.270). ERA were the most commonly prescribed monotherapy, with use marginally more frequent in those with PAH-only compared to PAH-ILD (PAH-only 68.8% vs. PAH-ILD 56.1%, *p* = 0.052). ERA and PDE5i were the most commonly prescribed combination therapy, with no difference between groups. Triple therapy was more common in those with PAH (PAH-ILD 0.9%, PAH-only 8.0%, *p* = 0.012). Lung transplantation was rare, but most common in ILD-only (*p* = 0.019).

#### Other disease manifestations

Peak C-reactive protein (CRP) and erythrocyte sedimentation rate (ESR) were highest in those with PAH-ILD and lowest in SSc-only (*p* < 0.001; Table 2). Previous SRC was more common in those with PAH-ILD (*p* = 0.040). Digital ulcers were most common in those with PAH-ILD or ILD-only (*p* < 0.001). People with ILD-only were more likely to have reflux symptoms (*p* = 0.022), whilst those with PAH-ILD or PAH-only more frequently had other symptoms of upper gastrointestinal dysmotility including dysphagia and vomiting (*p* < 0.02).

Peak Rodnan skin scores were highest in those with PAH-ILD and ILD-only, consistent with higher prevalence of dcSSc (*p* < 0.001). Non-hand skin ulcers were more common in those with ILD-only (*p* < 0.001), whilst painful calcinosis (requiring antibiotics or surgery) was more common in those with PAH-ILD (*p* < 0.001). Myositis was more common in those with PAH-ILD and ILD-only (*p* = 0.014), with higher peak CK levels in these groups (*p* < 0.002). Sicca symptoms were more common in PAH-only (*p* < 0.001). Haemoglobin levels were lower in those with PAH-ILD or PAH-only (*p* < 0.001).

#### SSc-specific treatment

Proton pump inhibitor (PPI) use was extremely common, especially in those with ILD-only and PAH-only (*p* < 0.001). Those with ILD (ILD-only or PAH-ILD) were more likely to have received immunosuppressive therapy, including prednisolone (*p* < 0.001), azathioprine (*p* < 0.001), mycophenolate (*p* < 0.001) and cyclophosphamide (*p* < 0.001; Table 2). Use of rituximab was uncommon but highest in PAH-ILD or ILD-only (*p* = 0.018), as was tocilizumab use (*p* = 0.018). People with ILD-only were most likely to have received methotrexate (*p* < 0.001) and hydroxychloroquine (*p* = 0.043). There were no differences in frequency of use of any immunosuppressant when PAH-ILD and ILD-only groups were compared (Table 2).

### Survival analyses

**Fig. 2:**
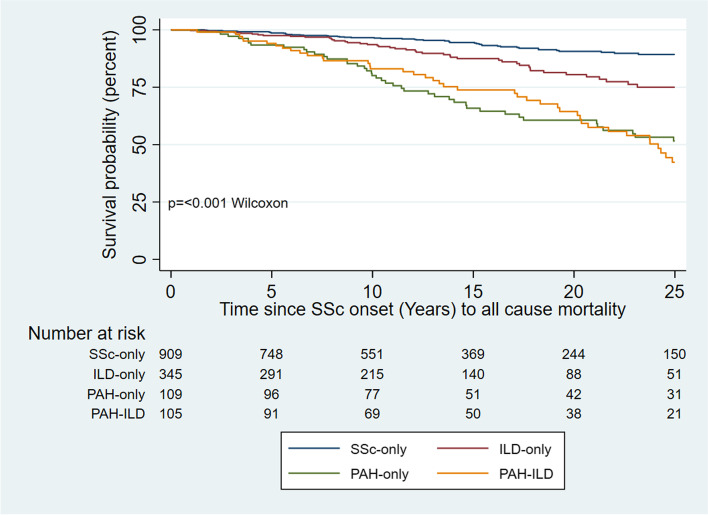
All-cause mortality from SSc onset. ILD, interstitial lung disease; PAH, pulmonary arterial hypertension; SSc, systemic sclerosis

**Fig. 3:**
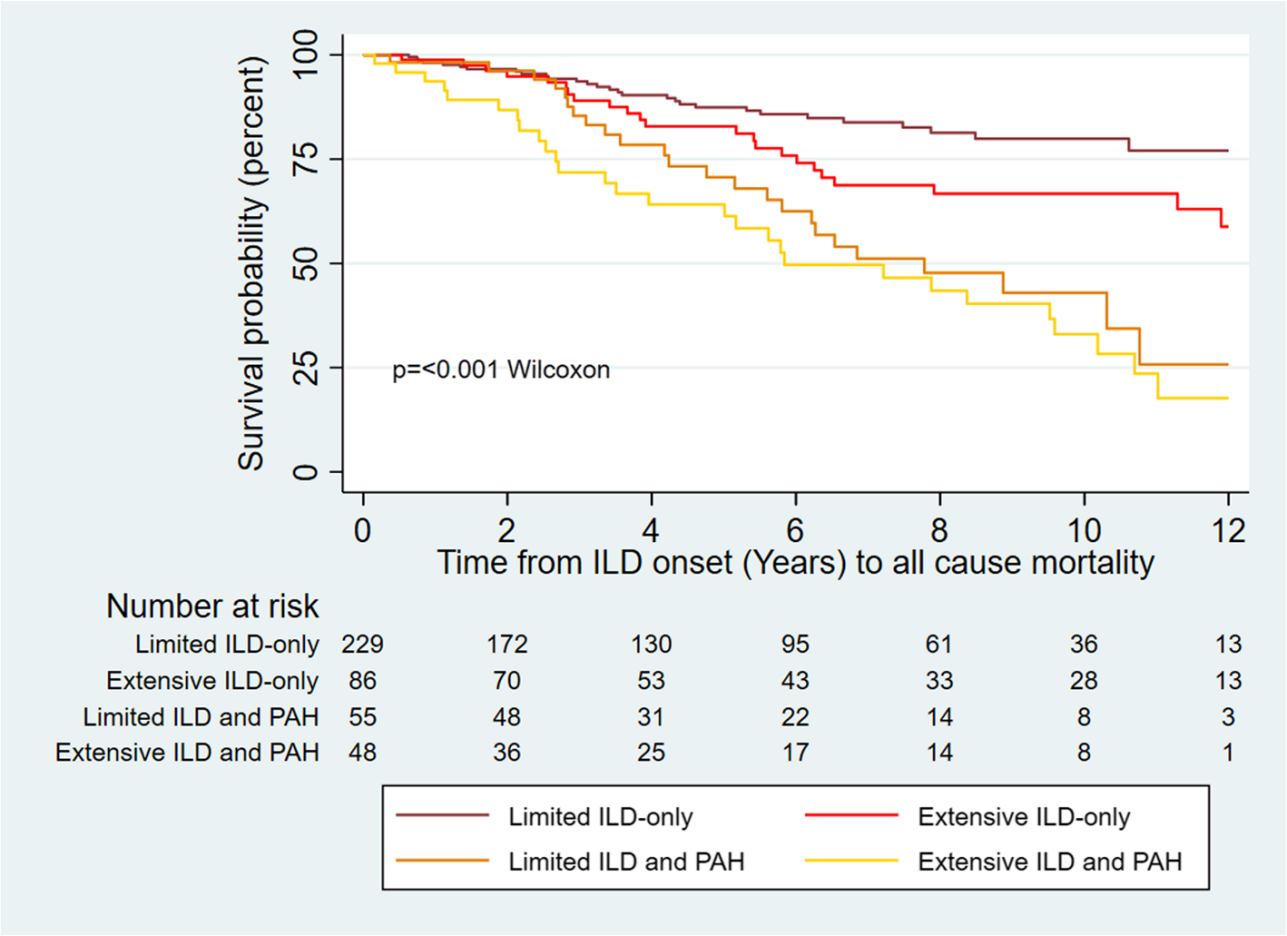
All-cause mortality from ILD onset in those with ILD. ILD, interstitial lung disease; PAH, pulmonary arterial hypertension; SSc, systemic sclerosis. ILD severity is defined as limited if < 20% involvement on HRCT or 20‒30% HRCT involvement with FVC ≥ 70%, or extensive if > 30% HRCT involvement, or 20‒30% HRCT involvement and FVC < 70%

Survival was significantly worse in those with PAH-ILD compared to SSc-only (*p* < 0.001, Fig. 2). However, the prognosis in those with PAH (PAH-only or PAH-ILD) was significantly worse than the prognosis of people with either ILD-only or SSc-only (*p* < 0.001, Fig. 2). After stratification by ILD severity, survival was poorest in those patients with PAH-ILD, with a graded relationship between severity of ILD and survival observed (*p* < 0.001, Fig. 3).

In a multivariable model of all-cause mortality, extensive ILD combined with PH conveyed the worst prognosis (HR = 5.68, 95% CI 3.51–9.17, *p* < 0.001), followed by those with PAH-only (HR = 4.30, 95% CI 2.95–6.27, *p* < 0.001), extensive ILD-only (HR 3.72, 95% CI 2.26–6.13, *p* < 0.001) and those with PH and limited ILD (HR = 2.60, 95% CI 1.59–4.24, *p* < 0.001; Table 3, univariate analyses in Supplementary Table S2). There was a difference in survival between those with SSc-only and limited ILD-only that did not reach statistical significance (HR = 1.52, 95% CI 0.96–2.42, *p* = 0.074; Table 3). Other significant risk factors for mortality were history of SRC (HR 2.60, 95% CI 1.48–4.56, *p* = 0.001) and older age at SSc onset (HR = 1.11, 95% CI 1.09–1.12, *p* < 0.001). Protective factors included ANA centromere positivity (HR = 0.65, 95% CI 0.48–0.91, *p* = 0.011) and female sex (HR = 0.61, 95% CI 0.43–0.86, *p* = 0.005).

**Table 3:** Multivariable model for survival from disease onset to death by ILD severity

Disease characteristic	HR	95% CI	*p*-value
Age at SSc onset (years)	1.11	1.09–1.12	< 0.001
ANA centromere positive	0.65	0.48–0.91	0.011
Female sex	0.61	0.43–0.86	0.005
History of renal crisis	2.60	1.48–4.56	0.001
ILD-only (limited)^a^	1.52	0.96–2.42	0.074
ILD-only (extensive)^a^	3.72	2.26–6.13	< 0.001
PAH	4.30	2.95–6.27	< 0.001
PAH and limited ILD^a^	2.60	1.59–4.24	< 0.001
PAH and extensive ILD^a^	5.68	3.51–9.17	< 0.001
Charlson Comorbidity Index Score	0.94	0.86–1.02	0.156

Disease onset is defined as the onset of the first non-Raynaud’s phenomenon symptom

*Abbreviations*: *ANA* anti-nuclear antibody, *ILD* interstitial lung disease, *PAH* pulmonary arterial hypertension

^a^ILD severity defined as limited if < 20% involvement on HRCT or 20–30% HRCT involvement with FVC ≥ 70%, or extensive if > 30% HRCT involvement, or 20–30% HRCT involvement and FVC < 70%

### Quality of life measures

Using GEE regression modelling, those with PAH-ILD were more than 3 times more likely than those with SSc-only to have a worse sHAQ score than the ASCS cohort median (*p* < 0.001), whilst those with PAH-only were twice as likely (*p* < 0.001) and those with ILD-only were 1.5 times as likely (*p* < 0.001; Table 4). Compared to those with SSc-only, those with PAH-ILD were more than 12 times more likely to have a worse PCS score than the overall SSc population (*p* < 0.001), whilst those with PAH-only were sevenfold more likely (*p* < 0.001), and those with ILD-only were twice as likely (*p* < 0.001; Table 4). There was no significant difference in SF-36 mental component summary (MCS) scores of the SF-36 between groups.

**Table 4:** Generalised estimating equation (GEE) regression analyses of health-related quality of life measures over time

Variable	Value	*N*^a^	OR	95% CI	*p*
**SHAQ scores**^b^					
Disease group	SSc-only	2943	.	.	.
	ILD-only	1213	1.54	1.27 to 1.87	< 0.001
	PAH	229	2.23	1.56 to 3.17	< 0.001
	PAH-ILD	181	3.41	2.26 to 5.14	< 0.001
**Physical component summary scores of the SF-36**^**c**^					
Disease group	SSc-only	2201	.	.	.
	ILD-only	899	2.13	1.61 to 2.80	< 0.001
	PAH	175	7.05	3.15 to 15.78	< 0.001
	PAH-ILD	132	12.48	4.11 to 37.91	< 0.001
**Mental component summary scores of the SF-36**^**c**^					
Disease group	SSc-only	2201	.	.	.
	ILD-only	899	1.06	0.86 to 1.31	0.586
	PAH	175	0.92	0.63 to 1.34	0.693
	PAH-ILD	132	1.26	0.83 to 1.91	0.248

*Abbreviations*: *CI* confidence interval, *GEE* generalised estimating equation, *ILD* interstitial lung disease, *OR* odds ratio, *PAH* pulmonary arterial hypertension, *SF-36* Short Form Survey 36, *SSc* systemic sclerosis, *sHAQ* Scleroderma Health Assessment Questionnaire

^a^Number of observations available for longitudinal analysis

^b^The odds of sHAQ score being greater (worse) than median sHAQ for ASCS population [13]

^c^The odds that the physical or mental component summary score being equal to, or below (worse than) the population average of 50

## Discussion

In a cohort of 1561 people with SSc, 6.9% had precapillary PH and ILD, 7.2% PAH-only, 23.9% had ILD-only and 62.1% SSc-only (Fig. 1). Individuals with PAH-ILD were more likely to be male, have dcSSc, higher inflammatory markers and an older age of SSc onset. People of Asian ethnicity more frequently had PAH-ILD or ILD-only. Those with PAH-only were more likely to have lcSSc. Serologically, ANA centromere was most common in those with PAH-only or SSc-only, whilst RNA polymerase-3 antibodies were most common in those with PAH-ILD.

Limited data exist in the wider literature to describe individuals with SSc who develop concurrent precapillary PH and ILD. We demonstrated a higher proportion of males in the PAH-ILD group which is consistent with other studies [10, 12]. In our cohort, people with both PAH-ILD and PAH-only were older at SSc onset; other data suggest that those with PAH-only may be older than those with ILD-associated PH [12]. We demonstrated a higher prevalence of dcSSc in those with PAH-ILD or ILD-only, although lcSSc was more common in those with PAH. Individuals of Asian ethnicity were more likely to develop PAH and ILD (8.9%) or ILD-only (11.8% vs. SSc-only 2.9%). This is consistent with other data demonstrating not only a higher prevalence of ILD in Asian populations [20] but also poorer survival of Asian individuals even in multivariable models adjusting for disease subclass, age of SSc onset and presence of PAH and ILD [20].

A diagnosis of either precapillary PH or ILD portends a worse prognosis than SSc-only. However, we identified that people with PAH-ILD and PAH-only have a significantly worse prognosis than those with ILD-only. In fact, whilst survival was better in those with SSc-only than limited ILD-only, this did not reach statistical significance. Other studies confirm the dominant importance of PAH in determining survival in PAH-ILD [9, 10], finding that the presence of concurrent ILD may not significantly impact survival in SSc-associated precapillary PH. Our study demonstrates that extensive ILD and precapillary PH confers a particularly poor prognosis. Those with PAH-ILD were more likely to have extensive ILD and a lower FVC than those with ILD-only. This is consistent with recent data demonstrating a higher FVC is associated with better survival in PAH-ILD [10]. Interestingly, whilst univariate analyses indicated worse prognosis with increasing ILD severity, our multivariable model suggested that PAH-ILD with limited ILD conferred a better prognosis than extensive ILD-only. Given our model assessed all-cause mortality, this may be due to the particularly high prevalence of dcSSc in extensive ILD-only (55.6%, vs. 32.7% in PAH and limited ILD), after adjustment for disease duration and ANA centromere positivity.

In this study, those with PAH-only and PAH-ILD were equally likely to receive monotherapy or dual therapy with PH-specific medications; however, triple therapy was more commonly prescribed in those with PAH-only. This is consistent with other cohorts [10]. PH-specific therapies in those with PAH-ILD are associated with improved WHO functional class, 6MWD and survival [21]. The lower prevalence of combination therapy for PAH in this study reflects the progression in funding of PAH treatment in Australia. Combination therapy for PAH was funded for reimbursed use on the Pharmaceutical Benefits Scheme only from October 2020 [22], with some patients self-funding combination therapy prior to this.

Patient-reported physical function and HRQoL were poorest in those patients with PAH-ILD. Whilst the negative impacts of either PH or ILD on HRQoL in SSc have previously been reported [6, 23, 24], the significant additive impact of concurrent PAH-ILD on patient-reported outcomes is a novel finding. Whilst both sHAQ and SF-36 PCS scores were worse in those with PAH-ILD, PAH-only or ILD-only than those with SSc-only, those with PAH-ILD had the worst scores and a 12-fold increased risk of below-average scores. This demonstrates that HRQoL is reduced in the presence of PH or ILD in addition to SSc, with the greatest reduction in those with PAH-ILD. There was no difference in SF-36 MCS scores, perhaps indicating that the psychological burden of SSc is significant regardless of associated complications, which is in keeping with previous findings in SSc-associated PAH [6].

To our knowledge, this is one of the largest studies investigating clinical features and survival of people with SSc with both precapillary PH and ILD and the only detailed exploration of HRQoL in this group. We have used prospectively collected data to perform a comprehensive analysis of disease features, serological profile and survival. Participants met clear RHC criteria for precapillary PH with cases reviewed where uncertainty existed by two study clinicians and were managed as PAH. Suggesting we have captured a group of precapillary PH and ILD overlap, we found no significant differences in mPAP, PAWP or PVR between those with PAH-ILD or PAH in our study, whilst other cohorts have identified higher mPAP and right atrial pressure in those with PAH-only compared to PAH-ILD [10]. Other limitations included that people in our study were generally recruited around 7 years after disease onset, meaning there may be a degree of ‘survivor bias’ as those with more aggressive disease and early mortality are less likely to survive to recruitment. This would likely underestimate any differences in survival between groups. In our dataset HRCT was not routinely performed but rather performed in response to PFT abnormalities, clinical symptoms or abnormal chest auscultation. This means that some cases of mild, limited ILD may have been missed given data suggesting lower sensitivity of PFTs compared to HRCT testing [25]. However, with longitudinal follow-up of our cohort, progressive, clinically significant cases of ILD were likely to be detected over time as symptoms, signs or PFT abnormalities progressed. Similarly, historically, we have not routinely performed other markers of PAH including NT-pro-BNP in our cohort. This reflects the longitudinal nature of the ASCS which commenced recruitment in 2007 and the evolution of clinical practice. Whilst it is possible we have underestimated the frequency of PH, given the close monitoring of symptoms, PFT and TTE and longitudinal follow-up of our cohort most cases are likely to be detected. Given the predominantly Caucasian population, these data are limited in their generalisability to other racial groups. Finally, PAH-ILD was relatively uncommon, limiting the statistical power of certain subgroup analyses.

## Conclusion

The prevalence of concurrent precapillary PH and ILD in the ASCS is 7%, with important clinical, serological and prognostic differences between SSc complicated by ILD, PAH-only and PAH-ILD. Precapillary PH appears to be a more important prognostic factor than ILD. However, within ILD alone, there is significant heterogeneity; those with limited ILD appear to have a prognosis more similar to SSc-only. Further data are required to better understand these high-risk groups and determine optimal treatment.

## Supplementary Information

**Additional file 1: Supplementary Table S1.** CCI score calculation. **Supplementary Table S2.** Univariate analyses for survival from disease onset* to death by ILD severity. **Supplementary Figure S1.** Flow diagram of included participants.

## Data Availability

The data underlying this article are available in the article and its online supplementary material.

## Abbreviations

6MWD: Six-minute walk distance
ACR: American College of Rheumatology
ANA: Antinuclear antibody
ANOVA: Analysis of variance
ASCS: Australia Scleroderma Cohort Study
CI: Confidence interval
CK: Creatinine kinase
CRP: C-reactive protein
dcSSc: Diffuse cutaneous systemic sclerosis
DLCO: Diffusing capacity for carbon monoxide
ENA: Extractable nuclear antigen
EULAR: European league against rheumatism
ERA: Endothelin receptor antagonist
ESR: Erythrocyte sedimentation rate
FVC: Forced vital capacity
GEE: Generalised estimating equation
HR: Hazard ratio
HRCT: High resolution computed tomography
HRQoL: Health-related quality of life
ILD: Interstitial lung disease
IQR: Interquartile range
lcSSc: Limited cutaneous systemic sclerosis
mmHg: Millimetres of mercury
mPAP: Mean pulmonary artery pressure
PAH: Pulmonary arterial hypertension
PAWP: Pulmonary arterial wedge pressure
PDE5i: Phosphodiesterase type 5 inhibitors
PCA: Prostacyclin analogues
PFT: Pulmonary function test
PH: Pulmonary hypertension
PRO: Patient-reported outcomes
PPI: Proton pump inhibitor
PVR: Pulmonary vascular resistance
RHC: Right heart catheter
RVSP: Right ventricular systolic pressure
SD: Standard deviation
SF-36: Short form 36 survey
SSc: Systemic sclerosis
SRC: Scleroderma renal crisis
TTE: Transthoracic echocardiogram
WHO: World Health Organization
