# Supplementary material for: Clinical characteristics and survival of pulmonary arterial hypertension with or without interstitial lung disease in systemic sclerosis
Source: Arthritis Res Ther. 2023 May 12;25:77. doi: 10.1186/s13075-023-03059-x (PMC10176744; doi:10.1186/s13075-023-03059-x)
Supplement: Supplementary file 1 — Additional file 1: Supplementary Table S1. CCI score calculation. Supplementary Table S2. Univariate analyses for survival from disease onset* to death by ILD severity. Supplementary Figure S1. Flow diagram of included participants. [file 13075_2023_3059_MOESM1_ESM.docx]

**Supplementary Electronic Material**

**Supplementary Table S1: CCI Score calculation**

| **Original Charlson Comorbidity Index** | | **Adaptation** | |
| --- | --- | --- | --- |
| Item | Score | Item | Score |
| Cerebrovascular disease | 1 | Patient-reported Stroke/TIA* | 1 |
| Congestive heart failure | 1 | LVEF≤50%* | 1 |
| COPD/Asthma | 1 | Patient-reported COPD or asthma | 1 |
| Dementia | 1 | Not recorded; excluded | N/A |
| Depression | 1 | Not recorded; excluded | N/A |
| Hypertension | 1 | Patient-reported hypertension* | 1 |
| Diabetes without end organ dysfunction | 1 | Patient-reported diabetes* | 1 |
| Diabetes with end organ damage | 2 | Not recorded; excluded | N/A |
| Liver disease - Mild | 1 | Not recorded; excluded | N/A |
| Liver disease – moderate or severe | 3 | Not recorded; excluded | N/A |
| Myocardial infarction | 1 | Patient-reported angina or myocardial infarction* | 1 |
| Peripheral vascular disease | 1 | Patient-reported peripheral vascular disease or treatments* | 1 |
| Rheumatic disease | 1 | Applicable to all patients with SSc | 1 |
| Peptic ulcer disease | 1 | Excluded; not recorded independently of other gastrointestinal SSc manifestations | N/A |
| Hemiplegia | 2 | Not recorded; excluded | N/A |
| Moderate to severe renal disease | 2 | Creatinine>265umol/L ever, or previous dialysis or renal transplantation* | 2 |
| Any tumour | 2 | Patient-reported malignancy (excluding NMSC) | 2 |
| Metastatic solid tumour | 6 | Not recorded; excluded | N/A |
| Skin ulcers or cellulitis | 2 | Patient-reported non-hand skin ulcers* | 2 |
| Takes warfarin | 1 | Warfarin or other anticoagulation | 1 |
| Leukaemia | 2 | Patient-reported leukaemia | 2 |
| Lymphoma | 2 | Patient-reported lymphoma | 2 |
| HIV/AIDS | 6 | Not recorded; excluded | N/A |
| **Maximum score** | **38** | **Maximum Score** | **19** |

Abbreviations: AIDS (acquired immunodeficiency syndrome), COPD (chronic obstructive pulmonary disease), HIV (human immunodeficiency virus), LVEF (left ventricular ejection fraction), NMSC (non-melanoma skin cancer), SSc (systemic sclerosis), TIA (transient ischaemic attack), umol/L (micromoles per litre)

# **Supplementary Table S2: Univariate analyses for survival from disease onset* to death by ILD severity**

| **Disease characteristic** | **HR** | **95% CI** | **p-value** |
| --- | --- | --- | --- |
| ANA centromere positive | 0.58 | 0.44-0.76 | <0.001 |
| ILD-only (limited)^1^ | 1.82 | 1.18-2.82 | 0.007 |
| ILD-only (extensive) ^1^ | 3.73 | 2.35-5.92 | <0.001 |
| PAH | 5.19 | 3.60-7.48 | <0.001 |
| PAH-ILD (limited ILD) ^1^ | 5.67 | 3.57-9.00 | <0.001 |
| PAH-ILD (extensive ILD) ^1^ | 8.11 | 5.22-12.59 | <0.001 |
| Female sex | 0.46 | 0.33-0.64 | <0.001 |
| History of renal crisis | 2.77 | 1.66-4.62 | <0.001 |
| Age at SSc onset (years) | 1.11 | 1.09-1.12 | <0.001 |
| Diffuse Cutaneous SSc | 1.99 | 1.50 – 2.63 | <0.001 |
| Charlson Comorbidity Index Score | 1.18 | 1.10 – 1.28 | <0.001 |

^1^ILD severity defined as limited if <20% involvement on HRCT or 20-30% HRCT involvement with FVC≥70%, or extensive if >30% HRCT involvement, or 20-30% HRCT involvement and FVC<70%. *Defined as the onset of the first non-Raynaud’s phenomenon symptom. Abbreviations: ANA (anti-nuclear antibody), ILD (interstitial lung disease), PAH (pulmonary arterial hypertension).

**Supplementary Figure S1: Flow diagram of included participants**


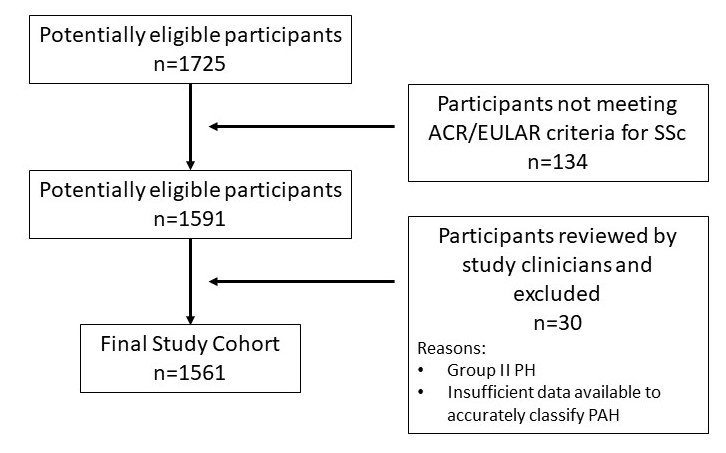


Abbreviations: ACR/EULAR (American college of Rheumatology, European League against Rheumatism), N (number), PAH (pulmonary arterial hypertension), PH (pulmonary hypertension).
